# Prognosis of Diabetic Peripheral Neuropathy via Decomposed Digital Volume Pulse from the Fingertip

**DOI:** 10.3390/e22070754

**Published:** 2020-07-09

**Authors:** Hai-Cheng Wei, Wen-Rui Hu, Na Ta, Ming-Xia Xiao, Xiao-Jing Tang, Hsien-Tsai Wu

**Affiliations:** 1 Basic Experimental Teaching & Engineering Training Center, North Minzu University, No. 204 North Wenchang Street, Yinchuan, Ningxia 750021, China; wei_hc@nun.edu.cn; 2 Laboratory of Intelligent Information and Big Data Processing of NingXia Province, North Minzu University, No. 204 North Wenchang Street, Yinchuan, Ningxia 750021, China; xiao_mx@nmu.edu.cn; 3 School of Electrical and Information Engineering, North Minzu University, No. 204 North Wenchang Street, Yinchuan, Ningxia 750021, China; 20187144@stu.nun.edu.cn (W.-R.H.); ta_na@nmu.edu.cn (N.T.); 4 School of Science, Ningxia Medical University, No. 1160 Shengli Street, Ningxia 750004, China; tangxj@nxmu.edu.cn; 5 Department of Electrical Engineering, Dong Hwa University, No. 1, Sec. 2, Da Hsueh Rd., Shoufeng, Hualien 97401, Taiwan

**Keywords:** diabetic peripheral neuropathy (DPN), percussion entropy index, baroreflex sensitivity (BRS), digital volume pulse (DVP), photoplethysmography (PPG), ensemble empirical mode decomposition (EEMD)

## Abstract

Diabetic peripheral neuropathy (DPN) is a very common neurological disorder in diabetic patients. This study presents a new percussion-based index for predicting DPN by decomposing digital volume pulse (DVP) signals from the fingertip. In this study, 130 subjects (50 individuals 44 to 89 years of age without diabetes and 80 patients 37 to 86 years of age with type 2 diabetes) were enrolled. After baseline measurement and blood tests, 25 diabetic patients developed DPN within the following five years. After removing high-frequency noise in the original DVP signals, the decomposed DVP signals were used for percussion entropy index (PEI_DVP_) computation. Effects of risk factors on the incidence of DPN in diabetic patients within five years of follow-up were tested using binary logistic regression analysis, controlling for age, waist circumference, low-density lipoprotein cholesterol, and the new index. Multivariate analysis showed that patients who did not develop DPN in the five-year period had higher PEI_DVP_ values than those with DPN, as determined by logistic regression model (PEI_DVP_: odds ratio 0.913, 95% CI 0.850 to 0.980). This study shows that PEI_DVP_ can be a major protective factor in relation to the studied binary outcome (i.e., DPN or not in diabetic patients five years after baseline measurement).

## 1. Introduction

Recently, many studies have reported that patients with type 2 diabetes could be at highly increased risk of developing atherosclerosis and autonomic nervous dysfunction [1,2,3,4]. Microvascular diseases are widespread among patients with long-term type 2 diabetes mellitus [5,6]. Generally, diabetic microvascular diseases are characterized by nerve damage caused by chronically high blood sugar and diabetes (e.g., diabetic peripheral neuropathy (DPN)) [7], exudate leakage from retinal small vessels (i.e., diabetic retinopathy), or persistent proteinuria and progressive decline in kidney function (i.e., diabetic nephropathy). DPN is a very common neurological disorder in diabetic patients [8,9]. The goals of caring for patients with type 2 diabetes mellitus are to reduce symptoms and to prevent, or at least slow down, the development of complications [10]. Glucose control in these patients undoubtedly has benefits for major microvascular endpoints; good glucose control does improve microvascular disease and should be achieved early and maintained over as long a period of time as possible [11,12,13,14]. On the other hand, early prediction of the signs of DPN through signal-analysis methods is urgently needed by diabetic patients and clinic doctors.

In recent years, the small-scale multiscale entropy index (MEI_RRI_) and the first proposed percussion entropy index (PEI_1st_) were developed to reflect autonomic function based on a nonlinear method for studying heart-rate variability (HRV) using only RR interval (RRI) datasets [15,16]. PEI_1st_ was also found to be suitable for analyzing diabetic patients [17]. In addition, percussion entropy–based indices are a generalized application of the method developed by Wei et al. (PEI_1st_) [18] and Xiao et al. (PEI_NEW_) [19] for specific time series, using amplitudes of successive digital volume pulse signals versus changes in RR intervals [15]. Wei et al. showed that PEI_1st_ can be used as a prognostic indicator with electrocardiography (ECG) and photoplethysmography (PPG) for future peripheral neuropathy in patients with type 2 diabetes [20]. However, using an ECG device is not convenient for many real-time applications. Considering surrogate data and cost reduction, a new percussion entropy index (PEI_PPI_) using only PPG (i.e., original amplitude series and peak-to-peak interval (PPI) series of the sixth decomposed intrinsic mode function (IMF6) from digital volume pulse (DVP)) was proposed to assess baroreflex sensitivity (BRS) complexity in elderly and diabetic patients with regard to type 2 diabetes associated autonomic dysfunction [21]. Moreover, PPG-derived DVP signals were further used for clinical applications in ubiquitous blood-pressure monitoring, congestive heart failure, and hypertension assessment [22,23,24]. However, it is not easy to filter out the high frequencies around peaks in the original DVP signals of diabetic patients, which can cause a big problem in error peak determination.

The investigation presented herein was conducted as follows: We hypothesized that the ensemble empirical mode decomposition (EEMD) method could be utilized for PPG-derived DVP signals to obtain two IMFs for decomposed DVP signals (IMF5 and IMF6) free of high-frequency noise [25]. Our second hypothesis was that a new lower value of percussion entropy index (PEI_DVP_) with the adoption of decomposed DVP-derived amplitude series and PPI series could estimate the prognosis of diabetic peripheral neuropathy for diabetic patients in the five years after baseline measurement.

This paper describes the use of decomposed DVP signals for percussion entropy analysis to assess the complexity of BRS for assessment of diabetic peripheral neuropathy prognosis. Descriptions of the study population, study procedure, PEI_DVP_ using synchronized PPI and Amp signals derived from decomposed DVP signals, and statistical analysis are presented in Section 2. Baseline characteristics of healthy and diabetic subjects; the failure of original DVPs to detect correct amplitudes in diabetic subjects; an assessment of agreement between amplitudes from original and decomposed DVPs of a healthy subject; a comparison of performance and computation time among PEI_DVP_, PEI_PPI_, PEI_1st_, and MEI_PPI_ to differentiate future peripheral neuropathy in type 2 diabetic patients; and the effects of risk factors associated with developing DPN using a logistic regression analysis method are presented in Section 3. All of our findings are discussed in Section 4. Finally, Section 5 concludes the paper.

## 2. Materials and Methods

### 2.1. Study Population

The study population consisted of 140 right-hand-dominant middle-aged subjects who underwent PPG and ECG examinations in the hospital from June 2009 to July 2011. Ten participants were excluded due to a history of atherosclerosis-associated complications, including permanent pacemaker implantation, coronary heart disease, heart failure, and ischemic stroke, leaving 130 subjects remaining in the study. In the study, diabetes mellitus was defined as a fasting glucose level higher than 126 mg/dL and/or a glycated hemoglobin (HbA1c) level greater than 6.5%. In total, 50 subjects were not diabetic patients (group 1, age range: 44–89 years), and 80 had type 2 diabetes. The diabetic patients were then divided into 2 groups: those who were diagnosed with type 2 diabetes without peripheral neuropathy within 5 years (group 2, age range: 44–86 years, n = 55), and those with peripheral neuropathy within 5 years (group 3, age range: 37–75 years, n = 25), all with HbA1c ≥ 6.5%.

In the present study, there were 11 diabetic patients in group 3 with good blood-glucose control (6.5% ≦ HbA1c < 8%), and 14 diabetic patients who originally had poor blood-glucose control (HbA1c ≧ 8%) [26]. During follow-up screening, DPN in type 2 diabetes patients was defined based on the presence of symptoms of numbness, tingling, or pain of distal extremities lasting for more than 3 months, along with a confirmed diagnosis by the clinic doctor in the same diabetes outpatient department through a neurophysiological study. For unbiased analysis, the subjects in the diabetic groups were age-controlled. This study was reviewed and approved by the Institutional Review Board of Hualien Hospital (Hualien City, Taiwan) and Ningxia Medical University Hospital (Yinchuan City, Ningxia, China).

### 2.2. Study Procedure

Anthropometric, demographic, and laboratory data for the analysis as well as medical history were obtained at the clinic visit. Each subject’s resting blood pressure was measured once from the left arm in a supine position using an oscillometric device (BP3AG1, Microlife, Taiwan). Total cholesterol, triglyceride, low-density and high-density lipoprotein cholesterol, fasting blood glucose, and glycosylated hemoglobin concentrations were obtained from blood samples after a 12 h fast. Caffeine-containing beverages and theophylline-containing drugs were forbidden for 12 h before each clinic visit. All measurements were taken in the morning (08:30–10:30). Moreover, to minimize latent erroneous readings from the PPG sensors arising from involuntary body vibrations of the test subjects and low environmental temperature, possibly resulting in constriction of the peripheral vessels, all subjects underwent blood sampling before data acquisition. All test subjects were allowed to relax in a supine position for 5 min in a quiet room with the temperature controlled at 26 ± 1 °C [26]. Data from the first 1000 cardiac cycles were used for analysis in this study.

### 2.3. Calculation of Percussion Entropy Index (PEI_DVP_) Using Synchronized {PPI} and {Amp} Signals

#### 2.3.1. Signal Processing for Percussion Entropy Indices

**Figure 1:**
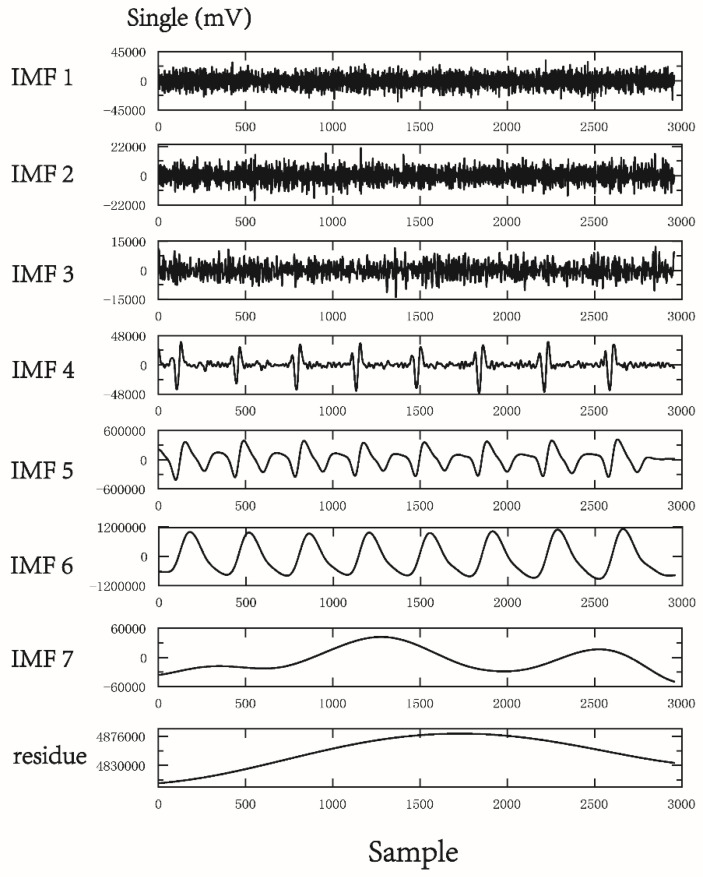
Representative illustration of original digital volume pulse (DVP) signals for group 1 subject; 7 intrinsic mode functions and residue were decomposed after ensemble empirical mode decomposition (EEMD). The amplitudes of intrinsic mode function (IMF) 5 and 6 were remarkably higher than those of IMF1–4. Moreover, fluctuations resembling systolic and diastolic peaks were noted in IMF5. IMF6 is sine wave-like, caused by the impact of the heartbeat on DVP signals from the fingertip, exhibiting a frequency close to that of the heart rate (i.e., 8 beats/6 (s) = 1.333 Hz).

**Figure 2:**
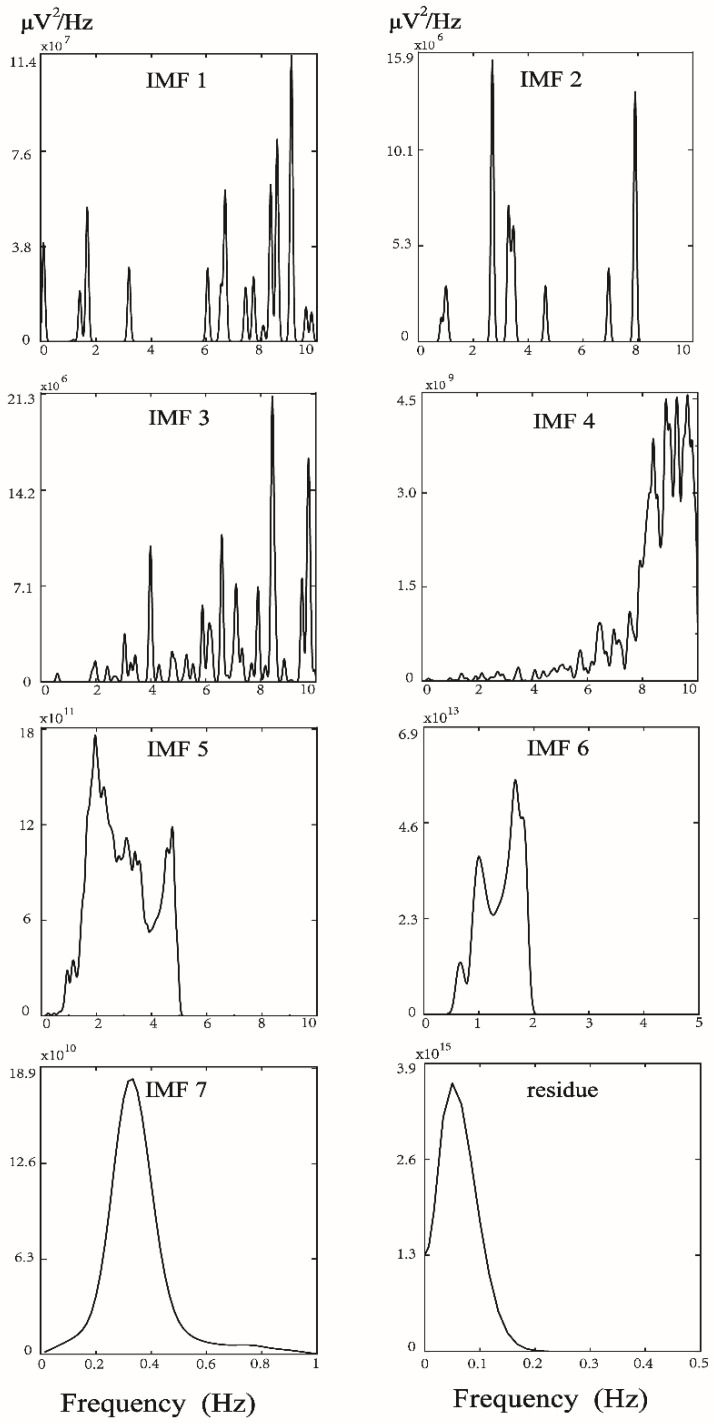
Marginal spectral density of 7 intrinsic mode functions and residue from a healthy subject, presented in Figure 1. Energy distribution analysis for IMF5 and IMF6 showed that their frequency distributions were as between 1–5 Hz. Hence, IMF5 and IMF6 were the noise-free component required in this study.

**Figure 3:**
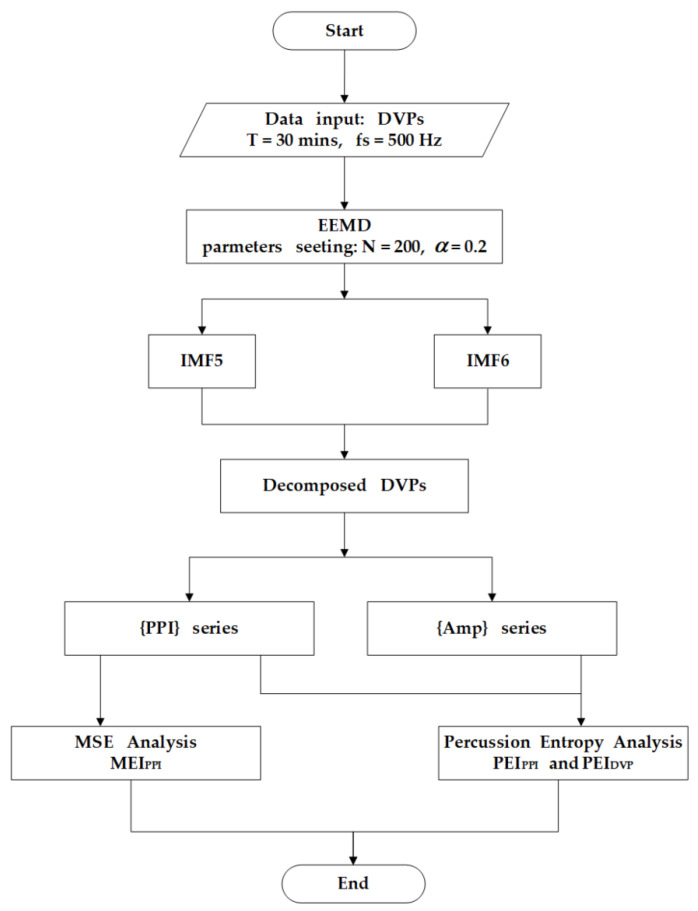
New percussion entropy index (PEI_DVP_) computation flowchart. Standard deviation of added noise was set as α = 0.2, and trial number was 200 for ensemble empirical mode decomposition (EEMD). As in [21], peak-to-peak interval of the sixth decomposed intrinsic mode function (IMF6) was used for multiscale entropy index (MEI**_PPI_**) calculation. After removing high-frequency noise in the original DVP signals, decomposed DVP signals (IMF5 and IMF6) were used for percussion entropy-based index computation.

The PPG infrared sensor was placed on the tip of the index finger of the subject’s dominant hand for data acquisition. After being run through a USB-based analog-to-digital converter (USB-6009 DAQ, National Instruments, Austin, TX, USA) with a sampling frequency of 500  Hz, the digitized signals were subsequently stored on a personal computer (PC) and analyzed using the MATLAB 7.7 software package (MathWorks, Natick, MA, USA) [26]. In each pulse cycle, ensemble empirical mode decomposition (EEMD) [27] was used to decompose the original DVP signals to many IMFs (Figure 1). As we know, EEMD interpretation heavily depends on “empirical” knowledge of the signals. In order to appropriately exploit the IMFs from original DVP signals, one has to be certain whether an IMF carries information relevant to the human system [28,29], from which selection of IMFs for further analysis can be made without doubt. Moreover, fluctuations resembling systolic and diastolic peaks were also noted in IMF5. IMF6 is sine wave-like, caused by the impact of the heartbeat on the digital volume pulse (DVP) signal from the fingertip, and exhibited a frequency close to that of the heart rate (Figure 1). Since the peaks of IMF5 and IMF6 are in phase and with larger amplitudes in IMFs from decomposed DVP in Figure 1, intuitively, the decomposed DVP signals (i.e., IMF5 + IMF6) can be regarded as noise-free original DVP signals. As a result, decomposed DVP signals were chosen for percussion analysis using the conventional method after decomposition of the original signal by EEMD (Figure 1 and Figure 2). We combined the fifth and sixth decomposed intrinsic mode functions (IMF5, IMF6) as decomposed DVP signals with high-frequency noise removed. Subsequently, the potential differences between peaks and valleys (defined as the lowest point after a peak) were defined as the pulse amplitudes of the decomposed DVP signals ({Amp}). In addition, {PPI} series were calculated from IMF6 (sine wave-like), caused by the impact of the heartbeat on the DVP, as addressed in [21]. The decomposed DVP and IMF6 signals had to be synchronized in the same way. Moreover, we checked whether peaks calculated from decomposed DVP signals were in phase with those from IMF6 or not. Finally, PEI_PPI_ [21], PEI_1st_ [18], and MEI_PPI_ [16] were computed and compared with the PEI_DVP_ proposed in this study (Figure 3).

#### 2.3.2. PEI_DVP_ Calculation from Decomposed DVP Signals

We tried to generate a new index using only DVP signals considering surrogate data and cost reduction in this study. That is, two specific time series were adopted: amplitudes of successive digital volume pulse signals, and changes in RR intervals of successive cardiac cycles from decomposed DVP signals.

- Synchronized {PPI} and {Amp} signals after EEMD
  {Amp} = {Amp_1_, Amp_2_,…, Amp_n_} for time series of decomposed DVP amplitude signals and {PPI} = {PPI_1_, PPI_2_,…, PPI_n_} for the PPI of IMF6 after EEMD were simultaneously synchronized for each subject.
  {Amp} = {Amp_1_, Amp_2_, Amp_3_,…, Amp_n_} (1)
  {PPI} = {PPI_1_, PPI_2_, PPI_3_,…, PPI_n_)} (2)
- Synchronized {U} and {V} signals for fluctuation patterns were computed as follows:
  {U} = {U_1_ U_2_ U_3 …_ U_n_}, U_i_ = 0, if Amp_(i+1)_ ≦ Amp_i_, or U_i_ = 1, if Amp_(i+1)_ > Amp_i_ (3)
  {V} = {V_1_ V_2_ V_3_ … V_n_}, V_i_ = 0, if PPI_(i+1)_ ≦ PPI_i_, or V_i_ = 1, if PPI_(i+1)_ > PPI_i_ (4)
- The percussion rate for each scale factor τ was computed as
  (5) $$P_{\tau }^{m}=\frac{1}{n-m-\tau +1}\sum_{i=1}^{n-m-\tau +1}\mathrm{count}\left(i\right).$$
  where m is the embedded dimension vectors and count(i) represents the match number between {U(i)} and {V(i + τ)}. A summation of all the numbers of matches (i.e., percussion number in Equation (5)) is thus obtained and divided by the total number of vectors of pattern, giving the “percussion rate,” where m is the impact point (e.g., m is set to be 2).
- As previous study [19], PEI_DVP_ calculation was as follows:
  (6) $$\mathrm{PEI}\;\left(m,n\tau \right)=\varphi^{m}\left(n\right)-\varphi^{m+1}\left(n\right).$$
  where $\varphi^{m}\left(n\right)=\ln\left(\sum_{\tau =1}^{n\tau }P_{\tau }^{m}\right)$, and nτ is the shift in number of scales considered.

**Figure 4:**
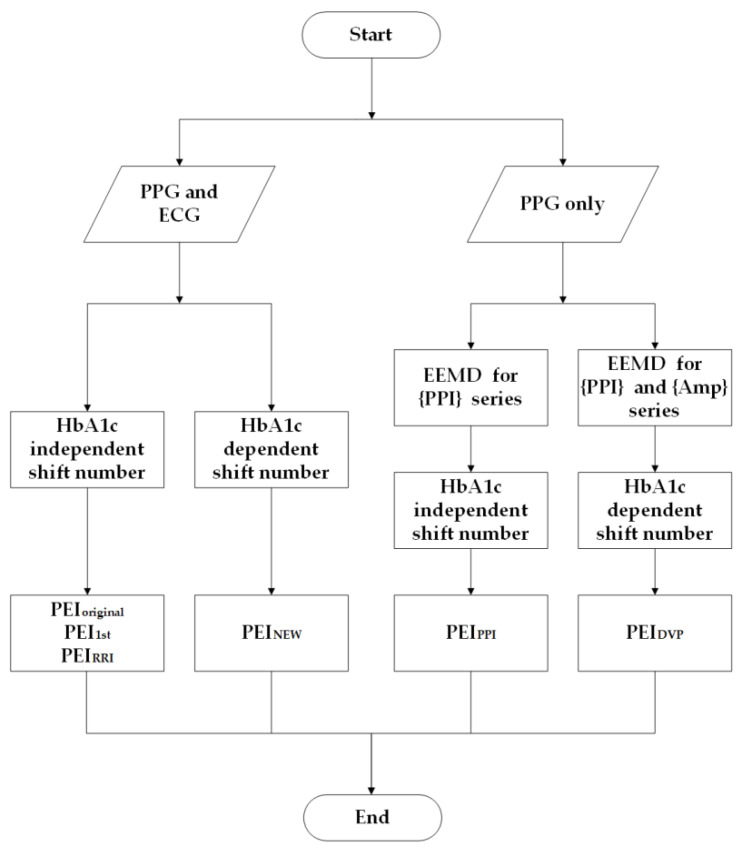
Flowchart of percussion entropy based index computations. Two synchronized photoplethysmography (PPG) pulse amplitude (Amp) and RR interval (RRI) series were acquired for PEI_1st_ (i.e., the same as PEI_original_ and PEI_RRI_) and PEI_NEW_. The difference between PEI_1st_ and PEI_NEW_ is in the shift number. Based on the previous findings, the baroreflex sensitivity regulation capability could differ among different HbA1c controls. For HbA1c dependent shift number, the shift number is set as 1 for subjects with HbA1c < 6.5, as 3 for subjects with 6.5 ≦ HbA1c < 8, and as 4 for subjects with HbA1c ≥ 8. Relatively, HbA1c independent shift number is always set as 5. On the other hand, PEI_DVP_ was calculated via decomposed digital volume pulses from the fingertip with HbA1c dependent shift number, whereas PEI_PPI_ was calculated via original {Amp} series and IMF6 with HbA1c independent shift number.

Wei et al. [18] chose nτ = 5 (i.e., HbA1c independent shift number) for PEI_1st_ in accordance with MEI_SS_. However, Xiao et al. proposed a speedy PEI index (PEI_NEW_) [19] and chose nτ = 1 for age-controlled healthy older subjects, nτ = 3 for diabetic subjects with good blood-sugar control (HbA1c < 8), and nτ = 4 for diabetic subjects with poor blood-sugar control (HbA1c ≥ 8) (i.e., HbA1c dependent shift number). Considering surrogate data and cost reduction (ECG was not used), PEIPPI was addressed in [21] with an HbA1c independent shift number. In this study, we followed the latter study in our choice of nτ with an HbA1c dependent shift number after EEMD for {PPI} and {Amp} from decomposed DVP signals, and PEIDVP was subsequently presented (Figure 4).

### 2.4. Statistical Analysis

Data in Table 1 and Table 2 are expressed as mean ± standard deviation. All tests were performed with SPSS version 14.0 for Windows (SPSS Inc., Chicago, IL, USA). Normality was assessed both visually (e.g., symmetry (skewness) and pointiness (kurtosis)) and with the Shapiro–Wilk test, provided by the SPSS software. We tested whether the data had approximately normal distribution, and data homoscedasticity was then verified. The comparisons of demographic, hemodynamic, anthropometric, and serum biochemical information of the test subjects were analyzed using two independent-sample t-tests with Bonferroni correction, and the differences between categorical variables were assessed using a chi-square test. The significance of differences in anthropometric, hemodynamic, and parametric values (PEI_PPI_, PEI_1st_, MEI**_PPI_**, and PEI_DVP_) among the groups was illustrated using independent sample t-tests with Bonferroni correction. Pearson’s correlation test in SPSS was also used to verify correlations between risk factors and the compared indices. A corrected p-value for multiple comparisons with a test-specific significance level of 0.017 was regarded as statistically significant. The effects of risk factors on the incidence of DPN in diabetic patients within 5 years of follow-up were tested by binary logistic regression analysis [28] with the Hosmer–Lemeshow goodness-of-fit test, controlling for age, waist circumference, low-density lipoprotein cholesterol, and the two PEI indices (PEI_PPI_ and PEI_DVP_).

## 3. Results

### 3.1. Testing of Healthy and Diabetic Subjects

Table 1 shows the baseline characteristics of the three groups. Compared with test subjects in healthy group 1, patients in group 2 were older and had higher body weight, waist circumference, body mass index, triglyceride (TG) levels, TG/HDL cholesterol ratio, fasting blood glucose, and glycosylated hemoglobin levels (all *p* < 0.001). It is worth mentioning that there were no notable differences between groups 2 and 3 in any demographic, anthropometric, hemodynamic, or serum biochemical information gathered from the test subjects (*p* > 0.017), as shown in Table 1.

### 3.2. Failure of Original DVPs to Detect Correct Amplitudes in Diabetic Patients

**Figure 5:**
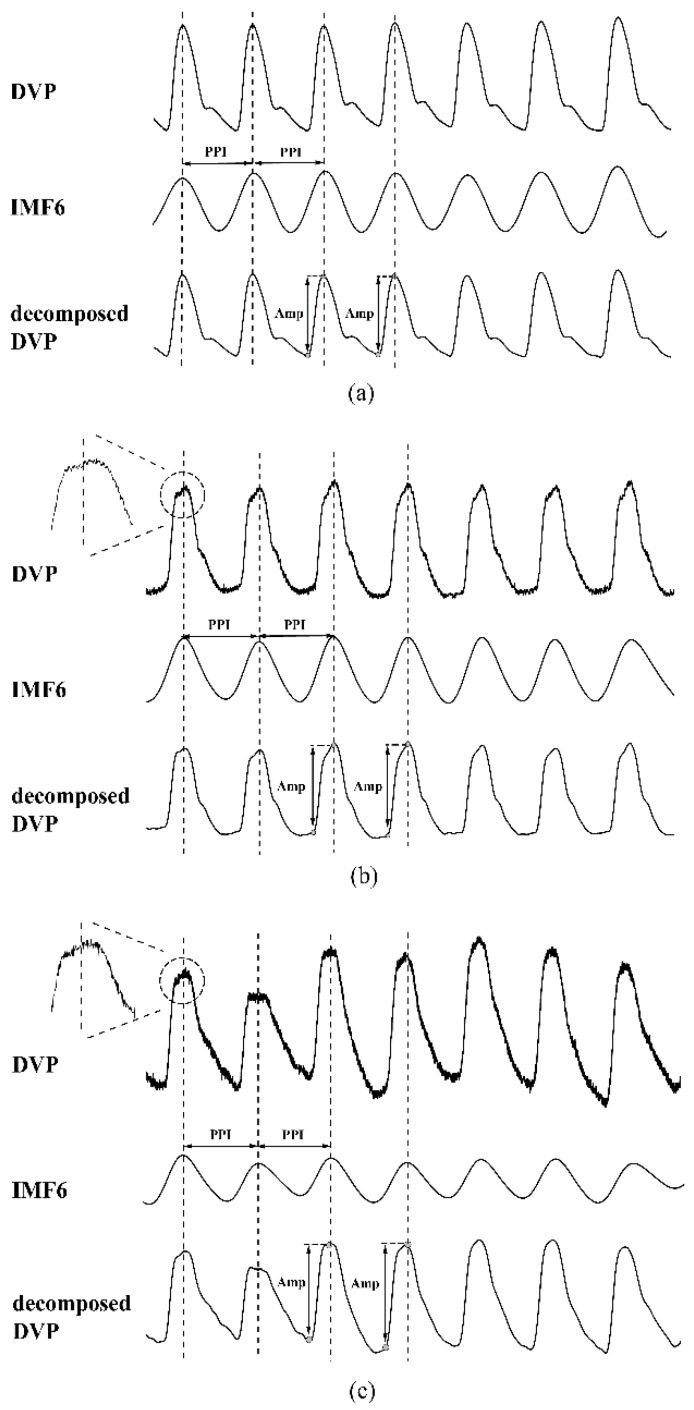
Digital volume pulse (DVP) signals, corresponding to decomposed sixth intrinsic mode function (IMF6), and decomposed DVP signals (IMF5 and IMF6) from one representative subject in each group: (**a**) subject A: healthy subject in group 1 (age: 49 years; WC: 88 cm; BMI: 23.8; HbA1c: 6.4%); (**b**) subject B: diabetic patient without peripheral neuropathy in group 2 (age: 57 years; WC: 90 cm; BMI: 25.2; HbA1c: 8.4%); (**c**) subject C: type 2 diabetic patient with peripheral neuropathy within 5 years in group 3 (age: 63 years; WC: 89 cm; BMI: 28.8; HbA1c: 10.1%). Peaks of DVP, IMF6, and decomposed DVP were in phase for subject A. It was difficult to calculate exact amplitudes from DVP for subjects B and C, due to high-frequency noise embedded in the original DVP signals. For all decomposed DVP signals, exact amplitudes could be calculated easily and accurately. Intuitively, the decomposed DVP signals can be regarded as noise-free original DVP signals for (**a**–**c**).

Figure 5 shows the failure of chaotic and subtle digital volume pulses measured at the fingertip of the dominant hand to detect the amplitudes for two diabetic subjects (Figure 5b,c), as compared with the stable peaks of a middle-aged nondiabetic subject (Figure 5a). The results can be attributed to interfering noise, such as that observed in patients with impaired peripheral circulation and respiration, and involuntary vibrations. By implementing EEMD, these noises were removed to obtain decomposed DVP signals (IMF5 and IMF6) for the calculation of exact amplitude series.

### 3.3. Assessment of Agreement between Amplitudes from Original and Decomposed DVPs

**Figure 6:**
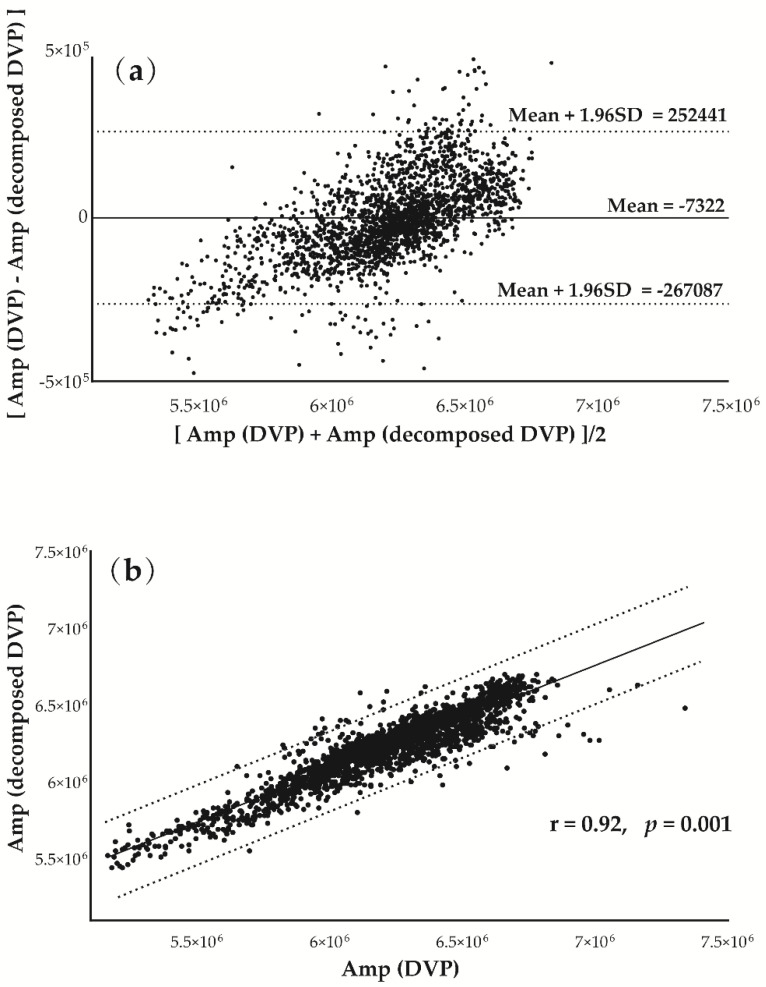
(**a**) Bland–Altman plot showing good agreement between two {Amp} sequences from DVP and decomposed DVP signals. (**b**) Positive correlation between {Amp} sequences from DVP and decomposed DVP signals for subject A in Figure 3 (r = 0.92, *p* = 0.001). Regression line describes the 95% confidence interval.

Figure 6 was prepared to verify the hypothesis that the decomposed DVP (IMF5 and IMF6) signals could replace the original DVP for amplitude series determination. Figure 6a shows Bland–Altman plots of these two measurements ({Amp} from decomposed and original DVP) for a subject from group 1. Good agreement was shown between the two measurements for the test subject. In addition, In Figure 6b, the strength of association between the variables is very high (r = 0.92), and the correlation coefficient is very highly significantly different from zero (*p* = 0.001).

### 3.4. Performance Comparison of PEI_DVP_, PEI_PPI_, PEI_1st_, and MEI_PPI_ to Differentiate Future Peripheral Neuropathy Prognoses in Type 2 Diabetic Patients

The results of comparing the three previous indices (MEI_PPI_, PEI_1st_, and PEI_PPI_) with the proposed PEI_DVP_ using Equation (6) in BRS evaluation of autonomic function assessment in the three groups are shown in Table 2.

MEI_PPI_ and PEI_1st_ were significantly higher in group 1 than group 2 (*p* < 0.001); however, there was not a statistically notable difference between groups 2 and 3. Relatively, PEI_DVP_ and PEI_PPI_ successfully discriminated between the test subjects in the three groups with statistically significant differences (all *p* < 0.017) (Table 2). Diabetic patients without DPN within five years (group 2) had significantly higher PEI_DVP_ than those who had developed DPN within five years (group 3) (0.63 ± 0.08 vs. 0.57 ± 0.08, *p* = 0.007, 95% CI of difference 0.016 to 0.095) (Table 2).

### 3.5. Effects of Risk Factors

#### 3.5.1. Correlation of Risk Factors with PEI_DVP_, PEI_PPI_, PEI_1st_, and MEI_PPI_

The associations of computational indices (MEI_PPI_, PEI_1st_, PEI_PPI_, and PEI_DVP_) with anthropometric (body weight and waist circumference) and serum biochemical (low-density and high-density lipoprotein cholesterol, triglycerides, triglyceride/HDL cholesterol ratio, fasting blood glucose, and glycated hemoglobin) factors of the three groups of test subjects were determined and analyzed using the Pearson correlation test in SPSS (Table 3). Compared to PEI_PPI_, PEI_DVP_ was associated with more risk factors.

#### 3.5.2. Effects of Risk Factors Associated with the Risk of Developing DPN

Multivariate analysis showed that subjects without DPN within five years (group 2) had higher PEI_DVP_ and PEI_PPI_ values than those with DPN within five years (group 3) (Table 2), as determined by logistic regression model [30] in SPSS (PEI_DVP_: odds ratio 0.913, 95% CI 0.850 to 0.980; PEI_PPI_: odds ratio 0.887, 95% CI 0.791 to 0.994).

Multivariate logistic regression analysis with a backward stepwise approach (likelihood ratio) in SPSS for incidence risk factors of DPN was conducted; the estimated model was with the Hosmer–Lemeshow test: χ^2^ = 10.03, degrees of freedom = 8, *p* = 0.263. R^2^ for logistic regression: Cox–Snell R^2^ = 0.243 and Nagelkerke R^2^ = 0.342. Overall percentage in classification table = 76%. There were four significant covariables in the fitted model: age (*p* = 0.011), waist circumference (*p* = 0.025), low-density lipoprotein (*p* = 0.031), and PEI_DVP_ (*p* = 0.017).

### 3.6. Computation Time for PEI_DVP_, PEI_PPI_, and PEI_1st_ in All Test Subjects

The computation times for the three indices for both nondiabetic and diabetic test subjects were obtained and compared. For this purpose, a personal computer (PC) was used, with specifications as follows: Hasee Notebook with Intel (R) Core (TM) i7-8750H CPU@2.200 GHz 2.20 GHz, Windows 10 Home. The computation program MATLAB R2016b (MathWorks Inc., Natick, MA, USA) was adopted. Two “tic” and “toc” instructions from the MATLAB package were used for the computation of CPU time.

Computation times for PEI_DVP_, PEI_PPI_, and PEI_1st_ for all subjects were computed and compared (Table 4). There are no differences in CPU time for PEI_DVP_ and PEI_PPI_. However, both PEI_DVP_ and PEI_PPI_ need decomposing time for EEMD computation, whereas PEI_1st_ can save EEMD decomposing time (Table 4).

## 4. Discussion

To quantify the asynchronism between two time series, many entropy-based analysis indices have been used in different research fields, including medicine, mechanics, civil engineering, environment, and finance [15]. To validate whether the dissimilarity of constitutive patterns in synchronized signals (i.e., {Amp} and {PPI} derived from decomposed DVP) in these two states could predict the severity of peripheral neuropathy impairment in this retrospective study, we looked backwards to examine exposure to suspected risk or protection factors in relation to an outcome established at the start of the study (i.e., diabetic patients who developed DPN five years after baseline measurement). As a result, decomposed DVP signals were chosen for percussion analysis using the conventional method after decomposition of the original signal by EEMD (Figure 1 and Figure 2). The decomposed DVP signals can be regarded as noise-free original DVP signals in Figure 5.

There were no statistically notable differences between the diabetic patients in groups 2 and 3 in anthropometric, hemodynamic, demographic, or serum biochemical parameters (all *p* > 0.017) (Table 1). That is, using these parameters, it would be very difficult to predict how many and which patients would develop peripheral neuropathy within five years. Previous studies [18,20] in which ECG and PPG signals were used proposed PEI_1st_ and presented its sensitivity in differentiating between diabetic subjects without DPN (e.g., with high PEI_1st_) and with DPN (e.g., with low PEI_1st_). However, an electrocardiogram (ECG) device is not convenient for some real-time applications. Considering cost reduction, PEI_PPI_ was addressed in [21], in which EEMD was used to separate noises to acquire refined DVP signals (IMF6) for exact PPI calculation in clinical applications. Nevertheless, accurate amplitude calculation from unstable DVP signals is still a problem, as there is always high-frequency noise around the peaks. After EEMD was conducted as proposed in [27,31], the DVP signals measured from fingertips were decomposed as many IMFs (the same IMF6 as used in [21]). In this study, IMF6 and IMF5 (fluctuations resembling systolic and diastolic waveforms in [25]) were added as noise-free decomposed DVP signals. We successfully identified the systolic and diastolic peaks proposed in [32] from the IMF5 component through EEMD for the diabetic patients (Figure 2 and Figure 3). Hence, this study looked at the results from PEI_DVP_, which showed statistically notable differences between all pairs of groups (group 1 versus group 2 versus group 3: 0.83 ± 0.18 versus 0.63 ± 0.08 versus 0.57 ± 0.08, all *p* < 0.017) (Table 2), which was consistent with the same finding in [33].

Poor glycemic control is a well-known risk factor for DPN [34,35]. In this study, out of the 25 diabetic patients in group 3, 11 had good blood-glucose control (HbA1c < 8%), and the other 14 had poor blood-glucose control (HbA1c ≧ 8%). Thus, 44% of DPN patients (11 out of 25) had good blood-glucose control at baseline measurement, but unfortunately developed further peripheral neuropathy within the study period. These results were consistent, and the associations of PEI_DVP_ with anthropometric (body weight and waist circumference) and serum biochemical (triglycerides, high-density lipoprotein cholesterol, fasting blood glucose, and glycated hemoglobin) parameters of all test subjects were statistically significant (Table 3). That is, another option (except for appropriate glucose control) exists for diabetic patients. Low PEI_DVP_ in a diabetic patient is recognized as the primary factor in prognosis (i.e., whether the patient is going to develop DPN in the near future), and is still considered the key component in clinical practice [36]. Previous study [36] demonstrated that a useful diagnosis is defined by patient prognosis. In this study, low PEI_DVP_ in a diabetic patient is recognized as the primary factor in prognosis (i.e., whether the patient is going to develop DPN in the near future). In addition, there is another important issue: we need to know the suspected risk or protective factors in relation to an outcome (e.g., diabetic patients developing DPN five years after baseline measurement) by using logistic regression analysis [30] in SPSS (PEI_DVP_: odds ratio 0.913, 95% CI 0.850 to 0.980; PEI_PPI_: odds ratio 0.887, 95% CI 0.791 to 0.994). The results confirmed again that both PEI_DVP_ and PEI_PPI_ were protective factors with regard to the binary outcome (i.e., whether diabetic patients developed DPN or not within five years after baseline measurement). Based on previous findings [19], the baroreflex sensitivity regulation capability could differ among different levels of HbA1c control. PEI_DVP_ was calculated via decomposed digital volume pulses from the fingertip with HbA1c dependent shift number, whereas PEI_PPI_ was calculated via original {Amp} series and IMF6 with HbA1c independent shift number (Figure 4). Accordingly, Figure 4 and Table 4 report percussion entropy based index computations. The computation times for PEI indices were determined by decomposing time for EEMD and CPU time for the index computations. The indices with HbA1c dependent shift number can save computation time (PEI_NEW_ and PEI_DVP_). The results in Table 4 show that CPU time for decomposed DVP signals by ensemble empirical mode decomposition would take longer. It goes without saying that the proposed method could be used in real time clinical applications in the near future.

This study still had some limitations. As a retrospective study, the number of test subjects enrolled was relatively small. Second, PEI_DVP_ was more complex for IMF5 and IMF6 computation than PEI_1st_, but the number of scales was smaller (five for PEI_1st_, three or four for PEI_DVP_ in Equation (6); Figure 4). Third, real-time processing was not possible for PEI_DVP_ computation because EEMD requires many operations. PEI_DVP_ information could not be provided immediately to the test subjects. In the future, using a real-time LabVIEW-based package could overcome this problem.

## 5. Conclusions

This study not only highlights that PPG-derived decomposed digital volume pulse signals can be freed of high-frequency noise by ensemble empirical mode decomposition and can be a major contributor to successfully determining amplitudes and stable peak-to-peak interval series (i.e., synchronized {Amp} and {PPI} signals), but also recommends the possible clinical application of a new percussion entropy index (PEI_DVP_) for use as a prognostic indicator of a protective factor for diabetic patients with peripheral neuropathy dysfunction.

## Figures and Tables

**Table 1:** Characteristics of the study population.

Parameter	Group 1	Group 2	Group 3
	N = 50	N = 55	N = 25
	Female/Male	Female/Male	Female/Male
	(27/23)	(18/37)	(10/15)
Age (years)	58.24 ± 9.38	67.20 ± 8.72 **	62.48 ± 8.45
Body height (cm)	160.74 ± 6.90	159.49 ± 6.88	163.72 ± 8.82
Body weight (kg)	61.71 ± 9.98	67.92 ± 9.73 **	71.76 ± 7.23
WC (cm)	83.72 ± 10.49	92.67 ± 8.43 **	95.48 ± 6.59
BMI (kg/m^2^)	23.85 ± 3.41	26.70 ± 3.59 **	26.94 ± 3.71
SBP (mmHg)	132.30 ± 36.37	126.45 ± 16.10	125.12 ± 30.33
DBP (mmHg)	81.22 ± 15.04	73.91 ± 9.89 *	71.84 ± 17.23
PP (mmHg)	52.60 ± 14.75	52.55 ± 12.37	53.28 ± 18.03
HDL (mg/dL)	51.82 ± 14.79	46.60 ± 15.98	39.28 ± 5.80
LDL (mg/dL)	121.15 ± 23.13	118.41 ± 38.38	105.28 ± 22.52
Cholesterol (mg/dL)	195.74 ± 40.65	179.30 ± 44.63	178.19 ± 28.57
Triglyceride (mg/dL)	99.71 ± 32.65	139.89 ± 65.41 **	160.82 ± 62.74
TG/HDL	2.06 ± 0.97	3.54 ±2.31 **	4.18 ± 2.38
FBS (mg/dL)	99.15 ± 17.88	148.22 ± 39.33 **	158.24 ± 54.18
HbA1c (%)	6.03 ± 1.37	7.87 ± 1.32 **	8.30 ± 1.45

Data are expressed as mean ± standard deviation. Group 1: healthy subjects; group 2: diabetic patients without peripheral neuropathy within 5 years; group 3: diabetic patients with peripheral neuropathy within 5 years. WC, waist circumference; BMI, body mass index; SBP, systolic blood pressure; DBP, diastolic blood pressure; PP, pulse pressure; HDL, high-density lipoprotein; LDL, low-density lipoprotein; TG/HDL, triglyceride/HDL ratio; FBS, fasting blood sugar; HbA1c, glycosylated hemoglobin. * *p* < 0.017 group 1 versus group 2, ** *p* < 0.001 group 1 versus group 2.

**Table 2:** Performance comparison among the three groups of test subjects for baroreflex sensitivity (BRS) and autonomic function assessment.

Parameters	Group 1 (N = 50)	Group 2 (N = 55)	Group 3 (N = 25)
MEI_PPI_	0.52 ± 0.18	0.37 ± 0.20 **	0.36 ± 0.19
PEI_1st_	0.71 ± 0.05	0.59 ± 0.11 **	0.61 ± 0.11
PEI_PPI_	0.68 ± 0.03	0.66 ± 0.04 *	0.63 ± 0.06 ^†^
PEI_DVP_	0.83 ± 0.18	0.63 ± 0.08 **	0.57 ± 0.08 ^†^

Data are expressed as mean ± standard deviation. Group 1: healthy subjects; group 2: diabetic patients without peripheral neuropathy within the study period; group 3: diabetic patients with peripheral neuropathy. MEI_PPI_: mean value of sample entropy on a scale from 1 to 5 using the {PPI} dataset only [16]. PEI_1st_: percussion entropy index using synchronized {RRI} and {Amp} from original DVPs [18]. PEI_PPI_: percussion entropy index using synchronized {PPI} and {Amp} from original DVPs [21]. PEI_DVP_: percussion entropy index using synchronized {PPI} and {Amp} from decomposed DVPs in this study. * *p* < 0.017 group 1 versus group 2, ** *p* < 0.001 group 1 versus group 2, ^†^ *p* < 0.017 group 2 versus group 3. *p*-value of unpaired student t-test less than 0.017 was noted as statistically significant in this study.

**Table 3:** Associations of anthropometric and serum biochemical risk factors with parameters in all test subjects.

Risk Factor	MEI_PPI_		PEI_1st_		PEI_PPI_		PEI_DVP_	
	r	*p*	r	*p*	r	*p*	r	*p*
Age (years)	−0.21	0.02	−0.14	0.09	−0.01	0.90	−0.13	0.12
BW (kg)	−0.05	0.52	−0.26	<0.001	−0.02	0.80	−0.30	<0.001
WC (cm)	−0.11	0.21	−0.33	<0.001	−0.07	0.46	−0.31	<0.001
LDL (mg/dL)	0.04	0.64	0.12	0.15	0.13	0.13	0.05	0.56
HDL (mg/dL)	0.09	0.31	0.19	0.03	0.03	0.70	0.16	0.05
TG (mg/dL)	−0.01	0.93	−0.30	<0.001	−0.04	0.61	−0.29	<0.001
TG/HDL	−0.02	0.76	−0.30	<0.001	−0.01	0.91	−0.28	<0.001
FBS (mg/dL)	−0.24	<0.001	−0.33	<0.001	−0.12	0.17	−0.37	<0.001
HbA1c (%)	−0.21	0.01	−0.38	<0.001	−0.19	0.02	−0.34	<0.001

MEI_PPI_: mean value of sample entropy on a scale from 1 to 5 using {PPI} dataset only [16]. PEI_1st_: percussion entropy index using synchronized {RRI} and {Amp} from original DVPs [18]. PEI_PPI_: percussion entropy index using synchronized {PPI} and {Amp} from original DVPs [21]. PEI_DVP_: percussion entropy index using synchronized {PPI} and {Amp} from decomposed DVPs. r: Pearson’s correlation coefficient is a measure of the linear correlation between index (e.g., MEI_PPI_, PEI_1st_, PEI_PPI_, and PEI_DVP_) and risk factor. It has a value between +1 and −1; 1 stands for total positive linear correlation, 0 means no linear correlation, and −1 signifies total negative linear correlation. *p*-values less than 0.05 were considered statistically significant.BW, body weight; WC, waist circumference; LDL, low-density lipoprotein; HDL, high-density lipoprotein; TG, triglyceride; TG/HDL, triglyceride/HDL cholesterol ratio; FBS, fasting blood sugar; HbA1c, glycosylated hemoglobin.

**Table 4:** Computation times for PEI_DVP_, PEI_PPI_, and PEI_1st_ in all test groups.

	Computation Time for Index		Group 1	Group 2	Group 3
**PEI**_1st_	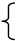	Average EEMD time (s)	0	0	0
		CPU time for index (ms)	21.29 ± 2.02	20.04 ± 1.61	20.39 ± 1.89
**PEI**_PPI_	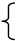	Average EEMD time (s)	7.06 ± 0.91	6.76 ± 0.75	6.67 ± 0.84
		CPU time for index (ms)	6.09 ± 0.81	17.55 ± 1.71 **	18.25 ± 1.25
**PEI**_DVP_	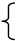	Average EEMD time (s)	7.06 ± 0.91	6.76 ± 0.75	6.67 ± 0.84
		CPU time for index (ms)	6.16 ± 0.86	17.69 ± 1.54 **	17.88 ± 1.64

PEI_1st_: percussion entropy index using synchronized {RRI} and {Amp} from original DVPs [18]. PEI_PPI_: percussion entropy index using synchronized {PPI} and {Amp} from original DVPs [21]. PEI_DVP_: percussion entropy index using synchronized {PPI} and {Amp} from decomposed DVPs. Group 1: healthy subjects; group 2: diabetic patients without peripheral neuropathy in study period; group 3: diabetic patients with peripheral neuropathy. ** *p* < 0.001: group 1 vs. group 2. Average EEMD time: CPU time for 3000 point DVP signals by ensemble empirical mode decomposition.
